# Endometrial receptivity defects MUC-1 and COX-2 polymorphisms in endometriosis

**DOI:** 10.25122/jml-2023-0192

**Published:** 2023-10

**Authors:** Uki Retno Budihastuti, Djaswadi Dasuki, Ahmad Hamim Sadewa, Totok Utoro

**Affiliations:** 1 Faculty of Medicine, Universitas Sebelas Maret, Surakarta, Jawa Tengah, Indonesia; 2 Dr. Moewardi General Hospital, Jawa Tengah, Indonesia; 3 Department of Obstetrics and Gynecology, Faculty of Medicine, Universitas Gadjah Mada, Yogyakarta, Indonesia; 4 Department of Biochemistry, Faculty of Medicine, Universitas Gadjah Mada, Yogyakarta, Indonesia; 5 Department of Anatomic Pathology, Faculty of Medicine, Universitas Gadjah Mada, Yogyakarta, Indonesia

**Keywords:** MUC-1, COX-2, polymorphism, endometrial receptivity, endometriosis, ARMS: Amplification refractory mutation system, BMI: Body mass index, COX-2: Cyclooxygenase-2, EDTA: Ethylene diamine tetraacetic acid, IL-11: Interleukin-11, MUC-1: Mucin 1, NSAID: Nonsteroidal anti-inflammatory drug, PCR-RFLP: Polymerase chain reaction-restriction fragment length polymorphism, PCR-SSPs: Polymerase chain reaction – sequence-specific-primers, PGE2: Prostaglandin E2, PGF-2α: Prostaglandin F-2α, PGHS-2: Prostaglandin endoperoxide H-synthase-2

## Abstract

The endometrium produces MUCIN-1 (MUC-1) and cyclooxygenase-2 (COX-2), which are essential for implantation. MUC-1 is required for adhesion, while COX-2 is necessary for decidualization. Variations or polymorphisms in MUC-1 and COX-2 can lead to changes in endometrial receptivity. This study investigated the relationship between MUC-1 and COX-2 polymorphisms and endometrial receptivity in endometriosis patients. Blood DNA samples were collected from 35 patients with endometriosis and 32 healthy patients between days 19 to 24 of their menstrual cycle (secretory phase). MUC-1 polymorphism was determined using the Amplification Refractory Mutation System (ARMS), and COX-2 gene polymorphism was assessed using Polymerase Chain Reaction-Restriction Fragment Length Polymorphism (PCR-RFLP). The frequency distribution of gene polymorphisms between the two groups was compared using bivariate analysis. There were seven genotypic combinations of MUC-1 and COX-2: AAGC; AAGG; GACC; GAGC; GAGG; GGGC; GGGG. The AAGC genotype combination test was significant, with an OR=6.43 (95% CI:1.09–7.62) and p=0.01. In conclusion, combining MUC-1 and COX-2 (AAGC) genotypes results in endometrial receptivity defects in endometriosis.

## INTRODUCTION

Endometriosis is a long-term inflammatory disease caused by estrogen, in which tissue similar to the endometrium is found outside the uterus in women. The most common symptoms include dysmenorrhea, chronic pelvic pain, and infertility [1-3]. The gold standard for endometriosis diagnosis is laparoscopy and biopsy, typically observed in Douglas's pouch, ovaries, uterosacral ligaments, and other small spaces or cavities [4-6]. Although genetic factors influence susceptibility to endometriosis, women with relatives with endometriosis are seven times more likely to be affected than women without a family history [2, 6]. Genomic changes in endometriosis promote alterations in proteomic character associated with endometrial receptivity. Endometrial receptivity is a physiological state that allows embryo implantation in the endometrium as the first step in the pregnancy process [7]. The presence of polymorphic genes, such as Cyclooxygenase-2 (COX-2) and Mucin-1 (MUC-1), can potentially cause proteomic changes. It is hoped that endometrial receptivity disorders in endometriosis patients can be detected through polymorphism analysis.

MUC-1 on the surface of endometrial cells expresses glycoprotein, which can hinder implantation. Some studies have demonstrated that implantation failure with a history of recurrent miscarriages is associated with MUC-1 genetic polymorphism [8]. The endometrium in endometriosis patients has steroidogenic factor 1 (SF-1) and aromatase, leading to increased estradiol production. Estradiol enhances the expression of COX-2 and the formation of Prostaglandin E2 (PGE2) [9]. COX-2 influences the synthesis of prostaglandins, particularly Prostaglandin F-2α (PGF-2α), in the luminal endometrial epithelium and plays a crucial role in the inflammatory process. Prostaglandin F2α (PGF2α) is a prostanoid in the family of synthesized and biologically active eicosanoid lipids resulting from a complex array of endothelial functions [10]. Pros-taglandin endoperoxide H-synthase-2 specially produces endome-trial prostaglandin products (PGHS)-2, also known as COX-2 [11, 12]. PGHS-2 converts arachidonic acid into prostaglandins as the primary precursor in generating various prostaglandins. PGE2 is involved in endometrial cell determination through interleukin-11 (IL-11) stimulation when IL-1 inhibits PGE2. Elevated PGE2 reduces decidualization during implantation [12]. In the proliferation phase, COX-2 expression is at its lowest point and remains high throughout the secretory phase [13]. This study aims to assess endometrial receptivity in endometriosis by evaluating differences in the frequencies of MUC-1 and COX-2 gene polymorphisms associated with endometrial receptivity.

## MATERIAL AND METHODS

### Research Subject

This study employed a case-control design, comparing 35 endometriosis patients experiencing infertility with 32 non-endometriosis patients. The case group comprised endometriosis patients confirmed by laparoscopy or laparotomy, subsequently validated through histopathological examination. In contrast, the control group consisted of women who underwent similar procedures but were not diagnosed with endometriosis. This latter group also encompassed women undergoing sterilization surgery and those with no clinical or historical indicators of endometriosis (specifically, those who were fertile, lacked histories of pelvic pain, dysmenorrhea, or dyspareunia, and exhibited standard gynecological clinical findings). The study was conducted from September 2010 to April 2012 and it included endometriosis patients with infertility who visited Dr. Sardjito Hospital, Dr. Moewardi Hospital, or the Islamic Hospital Klaten. Patients who underwent laparoscopy for endometriosis were included if they were between the 19^th^ and 24^th^ days of menstrual cycle. Patients using hormonal contraceptives, presenting malignancy, receiving medical treatment for endometriosis in the past six months, hormone replacement therapy, or using nonsteroidal anti-inflammatory drugs (NSAIDs) such as indomethacin in the past month were excluded. The dependent variables were MUC-1 and COX-2 expression, the independent variable was represented by the presence of endometriosis, and the external variables were age, body mass index (BMI), menstrual cycles, and age at menarche.

Hysterolaparoscopy was performed during the secretion phase (19^th^ to 24^th^ day) of the menstrual cycle. During this procedure, blood collection and endometrial tissue biopsies were conducted for endometriosis patients. Endometrial tissues from both groups underwent immunohistochemical examinations to assess MUC-1 and COX-2 expression. Concurrently, MUC-1 and COX-2 polymorphisms were analyzed in the venous blood samples of both endometriosis and control patients.

### DNA Isolation

To isolate DNA, 3-5 cubic centimeters (cc) of the subject's blood was placed in an anticoagulant tube containing Ethylene Diamine Tetraacetic Acid (EDTA). The sample was centrifuged at 3,500 rpm for 15 minutes to obtain the buffy coat layer. Next, 300 µL of the buffy coat was transferred to an Eppendorf tube, and 900 µL of cell lysis solution was added. The mixture was allowed to stand at room temperature for 10 minutes before being centrifuged at 13,000 rpm for 20 seconds. After removing the supernatant, 300 µL of cell nucleus lysis solution (for white blood cells) was added, vortexed, and the supernatant was removed again.

Subsequently, 100 µL of protein precipitation solution was added, vortexed for 20 seconds, and centrifuged at 13,000 rpm for 3 minutes to obtain the supernatant. The DNA supernatant was transferred to a new test tube containing 300 μL of isopropanol, inverted, and centrifuged at 13,000 rpm for one minute. The supernatant was discarded, and the sample was allowed to dry. Once dried, 300 µL of 70% ethanol was added, inverted, and centrifuged at 13,000 rpm for one minute. The supernatant was removed again, and the sample was dried using a hairdryer for 10-15 minutes. Lastly, 100 µL of DNA hydration solution was added and incubated at 65°C for one hour or overnight at 4°C. The DNA sample could then be identified by electrophoresis or stored at -20°C.

### MUC-1 Examination

The amplification-refractory mutation system (ARMS) was utilized to detect the presence of MUC-1 polymorphism. Amplification of the MUC-1 genes was performed using Polymerase Chain Reaction - Sequence-Specific Primers (PCR-SSPs) and primers forward 5'-CTA TGG GCA GAG AGA AGG-3' (primer 1) and reverse MUC-1 RI 5'-AGC TTG CAT GAC CAG AAC CC-3' (primer 2), MUC-1 RII 5'-AGC TTG CAT GAC CAG AAC CT-3' (primer 3), yielding a PCR product of 233 base pair (bp) in length [14]. The PCR reaction mixture contained DNA templates, Taq polymerase, dNTPs, MgCl2, primer combinations, and adequate distilled water. PCR conditions included an initial denaturation at 95°C for 10 minutes, followed by 35 cycles consisting of denaturation at 95°C for 45 seconds, annealing at 57°C for 45 seconds, and extension at 72°C for 45 seconds, concluding with a final extension at 72°C for seven minutes.

### COX-2 Examination

The analysis of COX-2 gene polymorphism was conducted using PCR-RFLP. The COX-2 gene was amplified with PCR using the forward primer 5'-GGC TGT ATA TCT GCT CTA TAT GC-3' and reverse primer 5'-CCG CTT TTG TCC ATC AG-3', yielding PCR products with a length of 306 bp [15]. The 306 bp PCR product was digested using the RFLP technique with the SsiI enzyme (AciI) # ER1791, sourced from Staphylococcus sciuri RFL1 Fermentas production. The digestion reaction mixture consisted of 5 μl of PCR product, buffer O 10x1 μl, 0.3 μl of SsiI enzyme (AciI), and 3.7 μl of distilled water. The reaction mixture was incubated at 37°C overnight. Digestion results were analyzed by electrophoresis using agarose gels and visualized with ethidium bromide under UV light.

In the healthy women group, the distribution of allele C was 7/64 (10.94%), and allele G was 57/64 (89.06%), while in the endometriosis group, allele C was 14/70 (20.00%), and allele G was 56/70 (80.00%). Based on these results, allele G was more common in healthy women, and allele C was more prevalent in endometriosis.

## RESULTS

### COX-2

The analysis of means for several variables in endometriosis and healthy women (Table 1) revealed that age, body mass index (BMI), and menstrual cycles had p>0.05, while age at menarche had p<0.05.

**Table 1:** Means of several variables in endometriosis and healthy women

Variable	Means of several		p-value
	Endometriosis (n=35)	Healthy Women (n=32)	
Age (years)	32.94±5.31	36.19±4.80	0.19
BMI (kg/m^2^)	21.43±1.57	22.55±3.17	0.96
Menstrual cycles (days)	27.77±2.33	27.75±2.35	0.88
Age at menarche (years)	15.14±2.19	13.34±1.35	0.01*

* : Significant; BMI: body mass index

**Figure 1:**
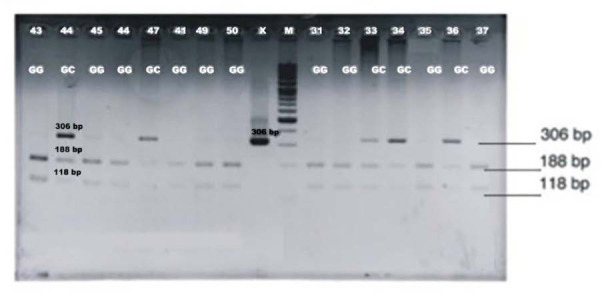
COX-2 electrogram results

Figure 1 displays the results of the COX-2 electrophoresis: CC remained uncut, GG was cut into two fragments with lengths of 188 and 118 bp, and GC produced three pieces of 306, 188, and 118 bp.

As shown in Table 2, the homozygous CC was found in one patient with endometriosis but not in healthy women. The relationship between the GC genotype and GG with COX-2 expression was not significantly different; however, the GC genotype was more prevalent in endometriosis than in healthy women.

**Table 2:** Distribution of genotype of COX-2

Genotypes/Alleles	Endometriosis	Healthy Women	OR (95%CI)	p-value
Genotypes	n=35	n=32		
CC	1 (2.86%)	0 (0.00%)	-	
GC	12 (34.28%)	7 (21.88%)	-	0.30
GG	22 (62.86%)	25 (78.12%)	-	
Alleles	n=70	n=64		
C	14 (20.00%)	7 (10.94%)	2.03 (0.77-5.37)	0.15
G	56 (80.00%)	57 (89.06%)	0.49 (0.19-1.30)	0.15

C - Cytosine, G - Guanine

### MUC-1

**Figure 2:**
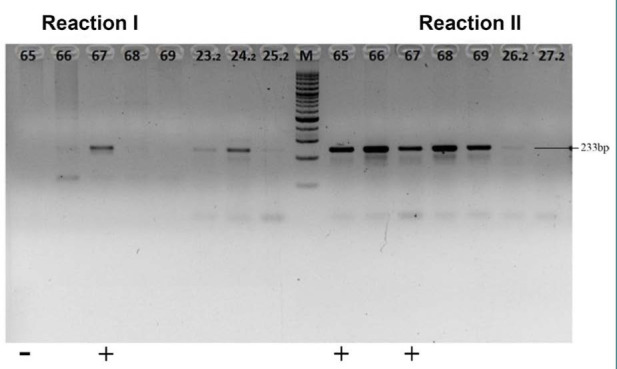
ARMS MUC-1 electrogram results

Figure 2 displays the results of the MUC-1 ARMS electrophoresis: Reaction I (RI) (+) and RII (-) indicate GG, RI (+) and RII (+) represent GA, RI (-) and RII (+) signify AA, and RI (-) and RII (-) represents an error (to be repeated).

Table 3 shows the allele distribution in the healthy women group, with A at 20/35 (57.14%) and G at 15/35 (42.86%). In contrast, the endometriosis group had an allele distribution of A at 50/99 (50.51%) and G at 49/99 (49.49%). Based on these results, both alleles A and G were more abundant in endometriosis than in healthy women.

**Table 3:** Distribution of genotype of MUC-1

Genotypes/Alleles	Endometriosis	Healthy women	OR (95%CI)	p-value
Genotypes	n=35	n=32		
GG	16 (45.71%)	18 (56.25%)	1.58 (0.08-29.16)	
GA	18 (51.43%)	13 (40.62%)	1.00 (Reference)	0.67
AA	1 (2.86%)	1 (3.13%)	1.41 (0.52-3.82)	
Alleles	n=99	n=35		
A	50 (50.51%)	20 (57.14%)	0.76 (0.35-1.67)	0.49
G	49 (49.49%)	15 (42.86%)	1.31 (0.60-2.85)	0.49

G – Guanine, A – Adenine

### Genotype MUC-1 and COX-2

Seven genotypes were combined in the MUC-1 and COX-2 groups: AAGC, AAGG, GACC, GAGC, GAGG, GGGC, and GGGG. In Table 4, the largest combined genotype was GAGG (58.33%) in endometriosis compared to AAGG (68.18%) in the control group. The differential test result indicated that AAGC had a significant endometriosis risk at p=0.01. The outcome met Hardy Weinberg's criteria. The conclusion is that AAGC carries the highest risk for endometriosis with OR=6.43; CI 95%=1.09-37.62; p=0.01. The combined genotype GAGG presents an en-dometriosis risk with OR=3.00; CI 95%=0.84-10.67; p=0.07, using AAGG as the reference for the normally combined genotype. Endometriosis is associated with the number of genes and their variants that clinically affect the pathogenesis of endometriosis. In the AAGC genotype combination test with OR 6.43 and p=0.01, the combination of MUC-1 and COX-2 genotypes alters endometrial receptivity. Similarly, for the GAGG group, which had an OR 3 times the risk of increased endometriosis incidence, the success of implantation was disrupted with a value of p=0.07. In the GC group, endometriosis was more prevalent than in the control group. GC genotypes may risk altering COX-2 expression and disrupting implantation during decidualization.

**Table 4:** Correlation combination genotype MUC-1 and COX-2 between endometriosis and healthy women

Karyotype Combination	Endometriosis	Healthy women	OR	CI 95%	p-value
AAGC	9 (75.00%)	3 (25.00%)	6.43	1.09 - 37.62	0.01*
AAGG^ref^	7 (31.82%)	15 (68.18%)	1.00	-	-
GACC	1 (100.00%)	0 (0.00%)	-	-	0.17
GAGC	3 (50.00%)	3 (50.00%)	2.14	0.32 - 14.20	0.41
GAGG	14 (58.33%)	10 (41.67%)	3.00	0.84 - 10.67	0.07
GGGC	0 (0.00%)	1 (100.00%)	0.00	-	0.51
GGGG	1 (100.00%)	0 (0.00%)	-	-	0.17

* : Significant; A – Adenine, G – Guanine, C - Cytosine

## DISCUSSION

Implantation begins with the blastocyst's opposition to the uterine epithelium. It is considered a pro-inflammatory reaction in which the ability of endometrial blood vessels can be significantly enhanced at attachment sites, mediated by Cyclooxygenase (COX) [16]. COX is an enzyme that acts as a rate-limiting agent for the metabolic conversion of arachidonic acid to pros-taglandin (PG) E2, influencing the implantation process. The pathophysiology of COX-2 expression in endometriosis is still not well understood, although numerous previous studies have reported COX-2 overexpression in endometriosis [1, 15, 16].

In endometrial tissue, MUC-1 expression increases from the proliferative phase to the secretory phase and decreases in the late secretory phase [17]. Increased estrogen levels influence the proliferative phase due to the growth of ovarian follicles, which can cause endometrioma regeneration through the proliferation of the epithelium, stroma, and blood vessel endothelium [14, 18]. The MUC-1 gene, located at 1q21-24 and encoding the MUC-1 protein, is now known to have two exon polymorphisms: a VNTR polymorphism in exon 2 and an A/G single nucleotide polymorphism (SNP) within exon 2 at site 568 [14].

Research by Kim *et al*. suggests that the MUC-1 genotype may not be correlated with endometriosis susceptibility. However, MUC4 polymorphisms are associated with the risk of endometriosis in Korean women [19]. These findings are con-sistent with those of Salazar *et al*., who reported that the presence of COX-2 polymorphisms results in impaired embryonic implantation [10]. In contrast, Kim *et al*. found that the -765C allele of the COX-2 gene was associated with a reduced risk of late-stage endometriosis [20]. COX-2 plays a significant regulatory role in the production of prostanoids related to trauma and inflammation. Therefore, we describe a functional COX-2 promoter polymorphism in the present study: -765G>C [15].

### Limitations

The limited sample size in our study can potentially introduce bias, as it might not adequately represent the broader population. Furthermore, such results may not be generalized to the wider population due to this inherent limitation.

## CONCLUSION

The combination of MUC-1 and COX-2 genotypes (AAGC) leads to defects in endometrial receptivity in endometriosis. There are differences in the frequency distribution of genotype combinations, with AAGC being more prevalent in endometriosis than in the control group.

Future studies on this topic would benefit from a more extensive sample to enhance the validity and generalizability of the results.

## Data Availability

The data sets used and analyzed during the current study are available from the corresponding author by reasonable request.

## Personal thanks

We thank the Department of Biochemistry, Faculty of Medicine, Universitas Gadjah Mada, for their permission and cooperation in conducting this research in their laboratory.
